# Supplementary material for: Low Surface Roughness Graphene Oxide Film Reduced with Aluminum Film Deposited by Magnetron Sputtering
Source: Nanomaterials (Basel). 2021 May 28;11(6):1428. doi: 10.3390/nano11061428 (PMC8227777; doi:10.3390/nano11061428)
Supplement: Supplementary file 1 [file nanomaterials-11-01428-s001.zip › nanomaterials-1233934-supplementary.pdf]

Support Information

Low Surface Roughness Graphene Oxide Film Reduced with Aluminum Film Deposited by Magnetron Sputtering

Xiaowei Fan [1], Xuguo Huai [2,*], Jie Wang [1], Tao Wang [1], Li-Chao Jing [1], Juncheng Liu [1], Hong-Zhang Geng [1,*]

1 Tianjin Key Laboratory of Advanced Fibers and Energy Storage, School of Material Science and Engineering, Tiangong University, Tianjin, 300387, China; xiaowei_fan@126.com (X.F.); wangjie01@visionox.com (J.W.); jinglctjpu@163.com (L.-C.J.); wangtao145411@foxmail.com (T.W.); jchliu@tiangong.edu.cn (J.L.)

2 Center for Engineering Internship and Training, Tiangong University, Tianjin, 300387, China

*Correspondence: huaixuguo@tiangong.edu.cn (X.H.); genghz@tiangong.edu.cn (H.-Z.G.); Tel.: +86-22-83955812 (H.-Z.G.); Fax: +86-22-83955055 (H.-Z.G.)

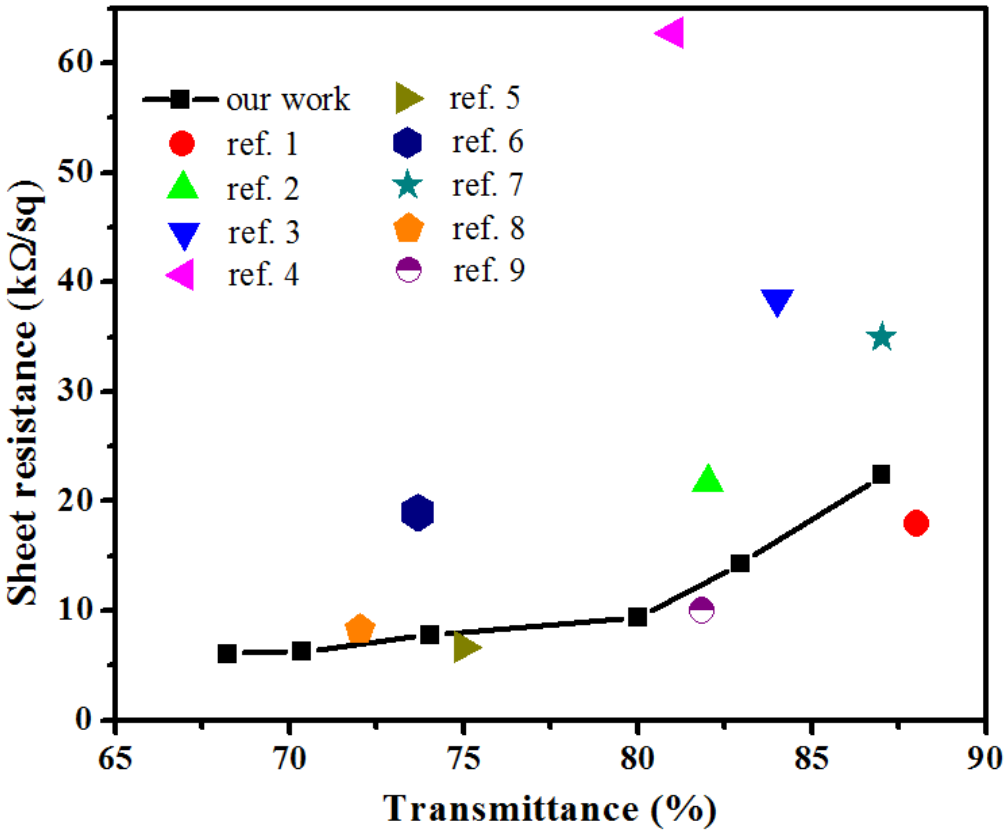

Figure S1. Comparison of transparency and conductivity of the rGO films deposited by different processes.

Reference

1. Kymakis, E.; Savva, K.; Stylianakis, M.M.; Fotakis, C. and Stratakis, E. Flexible organic photovoltaic cells with in situ nonthermal photoreduction of spin-coated graphene oxide electrodes. Adv. Funct. Mater., **2013**, *23*, 2742–2749.
2. Zhao, C.; Xing, L.; Xiang, J.; Cui, L.; Jiao, J.; Sai, H.; Li, Z. and Li, F. Formation of uniform reduced graphene oxide films on modified PET substrates using drop-casting method. Particuology, **2014**, *17*, 66–73.
3. Pham, V.H.; Pham, H.D.; Dang, T.T.; Hur, S.H.; Kim, E.J.; Kong, B.S.; Kim, S. and Chung, J.S. Chemical reduction of an aqueous suspension of graphene oxide by nascent hydrogen. J. Mater. Chem., **2012**, *22*, 10530–10536.
4. Ko, Y.U.; Cho, S.-r.; Choi, K.S.; Park, Y.; Kim, S.T.; Kim, N.H.; Kim, S.Y. and Chang, S.T. Microlitre scale solution processing for controlled, rapid fabrication of chemically derived graphene thin films. J. Mater. Chem. , **2012**, *22*, 3606–3613.
5. Ning, J.; Wang, J.; Li, X.; Qiu, T.; Luo, B.; Hao, L.; Liang, M.; Wang, B. and Zhi, J. A fast room-temperature strategy for direct reduction of graphene oxide films towards flexible transparent conductive films. J. Mater. Chem. A, **2014**, *2*, 10969–10973.
6. Kim, D.-Y.; Sinha-Ray, S.; Park, J.-J.; Lee, J.-G.; Cha, Y.-H.; Bae, S.-H.; Ahn, J.-H.; Jung, Y.C.; Kim, S.M.; Yarin, A.L. and et al. Self-healing reduced graphene oxide films by supersonic kinetic spraying. Adv. Funct. Mater. , **2014**, *24*, 4986–4995.
7. Domingues, S.H.; Kholmanov, I.N.; Kim, T.; Kim, J.; Tan, C.; Chou, H.; Alieva, Z.A.; Piner, R.; Zarbin, A.J.G. and Ruoff, R.S. Reduction of graphene oxide films on Al foil for hybrid transparent conductive film applications. Carbon, **2013**, *63*, 454–459.
8. Chen, F.; Liu, S.; Shen, J.; Wei, L.; Liu, A.; Chan-Park, M.B.; and Chen, Y. Ethanol-assisted graphene oxide-based thin film formation at pentane-water interface. Langmuir, **2011**, *27*, 9174–9181.
9. Shin, K.-Y.; Hong, J.-Y. and Jang, J. Flexible and transparent graphene films as acoustic actuator electrodes using inkjet printing. Chem. Commun., **2011**, *47*, 8527–8529.
